# Effect of Domestic Cooking of Hull-Less Barley Genotypes on Total Polyphenol Content and Antioxidant Activity

**DOI:** 10.3390/foods14152578

**Published:** 2025-07-23

**Authors:** Pavlína Podloucká, Ivana Polišenská, Ondřej Jirsa, Kateřina Vaculová

**Affiliations:** Agrotest Fyto, Ltd., Havlíčkova 2787/121, 76701 Kroměříž, Czech Republic; jirsa@vukrom.cz (O.J.); vaculova@vukrom.cz (K.V.)

**Keywords:** hull-less barley, domestic cooking, total polyphenol content, antioxidant activity

## Abstract

Barley is a good source of dietary fibre, vitamins, and minerals. Moreover, it is a source of polyphenols, which recently have been studied for their antioxidant properties. Barley generally is not eaten in its raw form, and the necessary processing influences the polyphenol content. This study evaluated the content of polyphenol compounds and antioxidant activity before and after thermal treatment typical for that carried out in households (i.e., boiling and subsequent microwave heating). Six genetic materials of hull-less barley were chosen for this study. The results showed that all tested barley genotypes were good sources of polyphenols. The studied thermal processes led to certain reductions in polyphenol content. The antioxidant activity of soluble phenolic compounds and the effects of heat treatment, as analysed by Trolox equivalent antioxidant activity (TEAC) and 2,2-diphenyl-1-picrylhydrazyl assay (DPPH) methods, differed. In the case of the DPPH method, the boiling and subsequent microwave heating indicated growth in antioxidant activity for almost all genotypes. When using the TEAC method, the results were not so clear, as the indicated activity both increased and declined. In the case of insoluble polyphenols, the antioxidant activity decreased for almost all genotypes regardless of the measurement method used.

## 1. Introduction

The term “whole grains” refers to foods containing all parts of the grain seeds (bran, germ, and endosperm) in their original proportions. Epidemiological studies have shown that consumption of whole grains provides a protective effect against chronic and civilization diseases, such as cardiovascular disease, diabetes, certain forms of cancer, and diseases connected with aging [1]. Although the exact mechanisms have not been completely determined, it is believed that the combined action of several components, such as dietary fibre and bioactive phytochemicals, including vitamins, minerals, and phenolic compounds, plays an important role in these health benefits [2,3].

Polyphenols are secondary metabolites of plants that have attracted the attention of food and medical researchers due to their strong in vitro and in vivo antioxidant activity, including abilities to scavenge free radicals, break radical chain reactions, and chelate metals. Antioxidant activity is mainly attributed to the structure of phenolic compounds, which possess at least one aromatic ring with bound hydroxyl groups. According to their structure, polyphenols are classified into several groups, including, among others, simple phenols, phenolic acids, flavonoids, lignans, and tannins. In cereals, the most important are phenolic acids whose structure is derived from either (i) hydroxybenzoic or (ii) hydroxycinnamic acid. In general, the hydroxycinnamic acid derivatives (such as ferulic and *p*-coumaric acids) are the predominant phenolic acids in cereals [4]. Phenolic compounds are not evenly distributed in cereal grains. Germ and bran fractions (aleurone and seed coat) contain higher concentrations [5,6]. Phenolic compounds in cereals exist in three primary forms: soluble free, soluble conjugated (linked with sugars), and insoluble bound (linked to cell wall components, such as cellulose, lignin, arabinoxylans, and structural proteins) [7]. The majority of phenolic acids in cereals (about 80%) are present in the insoluble form [2,8,9].

Barley (*Hordeum vulgare*) is the fourth most-produced grain worldwide, following wheat, maize, and rice [10]. It was one of the world’s earliest cultivated cereal crops, originating from the region of modern Israel and Jordan. Today, it is an adapted cereal crop which may be grown in diverse climatic conditions. Barley grain is a food providing beneficial effects on human health because it contains a large amount of dietary fibre, particularly β-glucans, as well as water- and fat-soluble vitamins (B-complex and vitamin E), minerals, and other bioactive phytochemicals, including polyphenols. These bioactive components are associated with several positive health influences, such as antioxidant, anti-inflammatory, antihyperlipidemic, antihypertensive, and antiproliferative effects, which are linked to prevention of the most chronic diseases and cancer [11]. In the past, barley was one of the basic staple cereals in the human diet, but it was gradually replaced by wheat, which has better technological and sensory properties (i.e., gluten strength, elasticity, texture, and taste) and is more suitable for bread-making. More recently, barley utilization has been primarily directed towards malting/brewing and animal feeding. Today, only 1–5% of barley produced is used directly for human consumption [11,12]. One of the problems complicating the utilization of barley as human food is the presence of tough fibrous hulls adhering strongly to the grains. Dehulling must precede consumption. Hulls, as indigestible hard casings of grains, must be removed by peeling and scouring before further processing, as a result of which the content of important bioactive substances, vitamins, and minerals in the final products could be significantly reduced. In recent decades, the breeding of barley intended for use in human nutrition has focused on the hull-less or “naked” varieties of barley. Such varieties have grains similar to those of wheat and so are more favourable for food production. Because the interest in healthy diets is increasing today, numerous barley-based food products are being made available (e.g., breakfast cereals, pasta, snack foods, etc.), as possible alternatives to wheat products. Barley in the form of groats is also used as a side dish, a pasta-like ingredient in soup, or a salad supplement.

Cereal grains are not generally consumed in their raw form but must undergo processing (peeling, scouring, grinding, and different kind of thermal processes), and that induces biological, physical, and chemical modifications. This naturally alters the sensory, nutritional, and textural properties of the final products. Consequently, these processes impact the contents of macro- and micronutrients and their bioavailability to the consuming organism. In the case of polyphenolic compounds, food processing may enhance or reduce their content, and this has implications for their bioactive properties and potential health benefits [13]. Grinding aside, thermal processing is the most common cereal process and takes various forms that include cooking, steaming, baking, roasting, extrusion cooking, microwave heating, and others. Thermal processing aims to improve taste and nutritive value by gelatinizing starches, denaturing proteins, increasing nutrient availability, as well as inactivating heat-labile toxic compounds and other enzyme inhibitors. Many studies have been published that deal with polyphenol content and the antioxidant activity of raw cereal grains [8,14,15,16,17,18,19]. It is known that the polyphenol content is influenced by cereal species [8,14,15,16], variety [17,18,19], growing location [19], and cultivation conditions [15]. In recent times, many researchers have been aware of the effect of processing on the content of phenolic compounds and their antioxidant activity. So, some studies and reviews describing the effects of cereal processing have been published [2,4,7,13,20,21,22,23,24,25,26,27,28,29,30,31,32,33,34,35,36,37,38,39,40,41,42,43,44]. In the case of barley, the studies have focused on malting [7], which is the most common process, as well as extrusion cooking [7,36,37] and baking [4,7]. Nevertheless, barley grains are also cooked and used in the form of groats. To our knowledge, there is still limited knowledge on the effect of boiling [35], which replicates the real use of barley seeds by common consumers, on the content of phenolic compounds and their antioxidant activity.

The main objective of this study was to investigate the effect of boiling and subsequent microwave heating on total phenol content and antioxidant activity in barley grains. The study was done on hull-less genotypes with standard and waxy types of starch. All chosen genotypes are intended for human nutrition and have been bred to contain higher levels of β-glucans, which are another component with a positive effect on human health. Among the many different culinary treatments, we chose for current study boiling, whereby the ratio of grains and water allowed complete absorption and subsequent microwave heating. Microwave heating has already become common in households. Unlike boiling, however, its effects on barley grains had not heretofore been studied.

## 2. Materials and Methods

### 2.1. Chemicals

All chemicals were analytical reagent grade. Methanol and water were HPLC grade. Folin–Ciocalteu reagent and Na_2_CO_3_ were obtained from Penta chemicals (Prague, Czech Republic); acetone, methanol, and water from Chem-Lab NV (Zedelgem, Belgium); HCl and NaOH from MicroCHEM (Pezinok, Slovak Republic). Gallic acid, DPPH, Trolox, K_2_S_2_O_8_, and ABTS were from Merck KGaA (Darmstadt, Germany).

### 2.2. Plant Material

For this study, six different hull-less spring barley genotypes intended for prospective human nutrition were selected. They were grown on experimental fields of Agrotest Fyto Ltd. in Kroměříž, Czech Republic, in the harvest year 2021. Two registered varieties (AF Lucius and AF Cesar) and one wild genetic source Nudimelanocrithon (NMC) with black-colour grains had standard starch composition, and three experimental breeding lines (KM 2975, KM 3189, KM 2551) had atypical amylose/amylopectin ratio. Genotypes with atypical amylose/amylopectin ratio are also known as “waxy” barley types characterised by low amylose content (0–5%) [45]. After harvest, the grains were stored in a dry and cold place. Barley genotypes were analysed in whole grain form (i.e., the grains were not peeled before grinding). To compare the effects of thermal treatment on phenolic content and antioxidant activity, barley grain was analysed in three forms: (i) as a raw material (grains ground just before the extraction using a Cyclone Sample Mill (UDY Corporation, Fort Collins, CO, USA), (ii) boiled, as well as (iii) boiled and subsequently microwave heated.

### 2.3. Cooking of the Barley

Barley grains (50 g) of each variety were placed into Erlenmeyer flasks and 60 mL of HLPC water was added to each. The barley-to-water ratio had been optimized in advance to ensure complete water absorption by barley grains during cooking. Grains were cooked under atmospheric pressure for 20 min to soft consistency. The flasks were covered by aluminium foil. After this time, the flasks were left to cool. Each boiled barley sample was then divided into 2 equal parts. One part was directly mixed by kitchen blender to pulp, and such prepared sample was used for extraction. The rest of the sample was left in a refrigerator at 4 °C for 2 days, after which the grains were heated in a household microwave oven (electric energy consumption 1150 W) at maximum power (700 W) for 3 min. This time had been chosen on the basis of previous testing of the microwaved oven used. Each sample was then mixed by blender to pulp and used for extraction.

### 2.4. Extraction

The procedure used for the polyphenol extraction was slightly modified from the procedure published by Podloucká et al. [46]. In test tubes, 0.5 g of ground grains or pulp from boiled grains was extracted using 10 mL of 80% acetone. Pulp of boiled grains was homogenised for 1 min by Ultra-Turrax T8 homogeniser (IKA-Werke GmbH & Co., KG, Staufen, Germany). The tubes were incubated overnight at ambient temperature in darkness. During this time, the mixtures were vortexed several times. The next day, the samples were centrifuged (10 min, 9900 rpm) by MPW-350R centrifuge (MPW Med. Instruments, Warsaw, Poland), and supernatants were decanted and used for analysis of the polyphenol content of soluble compounds and their antioxidant activity. The pellets in tubes were diluted in 10 mL of alkaline methanol (3:1, 4 mol/L NaOH and methanol), incubated at 80 °C for 120 min, and vortexed every 30 min. After that, the mixtures were cooled to ambient temperature and were neutralized by acidified methanol (3:1, 4 mol/L HCl and methanol). Mixtures were refilled up to 25 mL. This was followed by centrifugation for 10 min, and supernatants were used to analyse the total phenol content of insoluble compounds and their antioxidant activity.

### 2.5. Determination of Total Polyphenol Content

Total polyphenol content (TPC) was determined by modified Folin–Ciocalteu spectrophotometric assay, which is based on reduction of phosphowolframate–phosphomolybdate complex by phenols [47,48]. To 0.5 mL of sample extract in a test tube was added 0.25 mL of Folin–Ciocalteu reagent, then 8 mL of HLPC water and 1.25 mL of 20% (*w*/*w*) Na_2_CO_3_. The test tube was shaken and allowed to stand in darkness and at room temperature for 2 h. The absorbance was measured twice for each sample by Carl Zeiss Jena Spekol 11 spectrophotometer (Carl Zeiss, Oberkochen, Germany) at 760 nm against blank (0.5 mL of HLPC water was used instead of sample). Gallic acid was used as a standard for calibration curve construction. Due to the differences in moisture content in raw and cooked samples, all results were expressed on a dry-weight basis. Dry matter was determined gravimetrically, when sample was dried for 90 min at 130 °C (Table S1). The results are expressed as micrograms of gallic acid equivalent (GAE) per 1 g of the dried matter (DM). Four replications were carried out for each sample.

### 2.6. Determination of DPPH Scavenging Activity

The DPPH assay was measured according to the methods of Brand-Williams et al. (1995), with some modifications [49]. The stock solution was prepared by dissolving 24 mg of DPPH (2,2-diphenyl-1-pycrylhydrazyl radical) with methanol (100 mL) and was sonicated for 5 min. The working solution was obtained by refilling 10 mL of the stock solution with 50 mL of methanol to obtain a 1.1 absorbance at 515 nm. Fresh solution was prepared for each assay. The quantity 0.2 mL of grain extract was allowed to react with 3.8 mL of the working DPPH solution and left in darkness and at ambient temperature for 2 h. The absorbance was measured twice for each sample at 515 nm against methanol, which served as blank. Trolox was used as a standard for calibration curve, and the results are expressed as micrograms of Trolox equivalent (TE) per 1 g of the dried matter. Four replications were carried out for each sample.

### 2.7. Determination of Trolox Equivalent Antioxidant Activity

To measure Trolox equivalent antioxidant activity (TEAC), the procedure followed the method of Arnao et al. (2001), with some modifications [50]. The stock solution was prepared by mixing 50 mL of 3.73 mM ABTS (2, 2′-Azinobis (3-ethylbenzothiazoline-6-sulfonic acid) diammonium salt) solution with 1 mL of 59.9 mM K_2_S_2_O_8_ (potassium persulfate) solution and allowing these to react for 12–16 h in darkness at ambient temperature. The working solution was prepared by diluting 1 mL of stock ABTS solution with 45 mL of methanol to obtain an absorbance of 0.7 at 734 nm. Fresh solution was prepared for each assay. The 50 µL of extract was allowed to react with 4 mL of working solution and left to stand in darkness and at ambient temperature for 30 min. The absorbance was measured twice at 734 nm against methanol, which served as blank. Trolox was used as a standard, and results are expressed as micrograms of Trolox equivalent per 1 g of dried matter. Four replications were carried out for each sample.

### 2.8. Data Analysis

The TPC, DPPH scavenging activity, and TEAC were expressed as mean values from four replications ± standard deviation. All calculations were performed using the TIBCO Statistica 14.0 software package (TIBCO, San Ramon, CA, USA). The significance level was set at *p* ≤ 0.05 unless stated otherwise.

## 3. Results and Discussion

### 3.1. Total Phenolic Content (TPC)

To study the effect of boiling and subsequent microwave heating on the barley’s phenolic content, the unprocessed grains were first analysed. The phenolic compounds are considered to be the most important source of antioxidant activity in barley and exist in soluble (free and conjugated), and insoluble bound forms [8]. Soluble polyphenols were extracted in 80% acetone, insoluble polyphenols were released by alkali hydrolysis, and the total was expressed as a sum of both. The results are shown in Table 1.

Substantial amounts of total phenolics were detected in all studied barley genotypes. In the case of soluble phenols, small deviations were observed between individual genotypes. The mean TPC value was 2.24 mg GAE/g DM. Four of the six genotypes (i.e., all waxy-type lines and variety AF Cesar) were very close to this value, while variety AF Lucius had the highest and NMC the lowest value at 2.52 and 2.04 mg GAE/g DM, respectively. Such TPC values are in very good agreement with those published by Zhao et al. (2008) [51], who studied 14 malting barley cultivars and also used 80% acetone as a solvent for extraction. Their results were in a range from 2.17 to 2.56 mg GAE/g DM. That the TPC value for black genotype NMC was the lowest in the current study is not consistent with a study published by Abdel-Aal et al. (2012) [52], who stated that black varieties had higher contents of free phenolics compared with yellow varieties. Lee et al. (2010) [53] reported that waxy-type barley genotypes had higher TPC compared to non-waxy types. This also is not in agreement with the obtained results, wherein both waxy and non-waxy barley genotypes exhibited comparable contents of soluble phenols.

Regarding the insoluble polyphenols, their contents were comparable in all genotypes except for NMC, which yielded a significantly higher content value of 11.51 mg GAE/g DM versus the mean value for the other genotypes of 8.54 mg GAE/g DM. Such high values correspond to about 79% (for NMC 84%) of the total phenolic content. High insoluble phenolic content values reflect the fact that the majority of phenolic compounds are bound to the cell wall components of cereals grains. This is in agreement with data published by Adom et al. (2002) [8], who found that the major portion of phenolics in grains was in the bound form and measured 85%, 75%, and 62% for maize, oat or wheat, and rice, respectively. Bound phenolic compounds play an important antioxidant role in the gastrointestinal tract, where some could be released in part during the stomach and intestinal digestion, while others can be released by the action of colonic microorganisms [54,55].

If the sum of both soluble and insoluble phenols is considered, the highest phenol content was obtained for NMC (13.55 mg GAE/g DM) and the lowest for the experimental genotype KM 3189 (10.42 mg GAE/g DM).

Cereal-based food is usually consumed after thermal processing. Due to the fact that in this study we wanted to imitate the common preparation of barley grains in an ordinary household, grains were first boiled and then heated in a microwave oven. Surprisingly, the boiling for 20 min caused only minimal differences in the content values of both the soluble and insoluble polyphenols (Table 1 and Table S2).

Regarding the soluble phenolic content, slight increases were registered in two genotypes (AF Lucius and NMC), while the remaining genotypes showed decreases. However, these differences were not statistically significant. The highest value was registered for AF Lucius (2.55 mg GAE/g DM) and the lowest for AF Cesar (2.03 mg GAE/g DM). Cultivar AF Cesar had, at the same time, the highest loss of soluble phenolic content (9%). The remaining genotypes had mean values of soluble phenolic content varying around 2.13 mg GAE/g DM and losses of soluble phenolic compounds after boiling that varied near 4%.

As was mentioned in the introduction, there are not many studies dealing with the effect of boiling on the content of polyphenols in barley grains with which the results of this study can be compared. Gallegos-Infante et al. (2010) [35] studied thermal processing of the Mexican hulled barley cultivar Esmeralda intended for malting, and they used 70% acetone as a solvent for extraction. Their results showed that cooking resulted in an increase in the free phenolic content values compared to unprocessed barley. This increase was explained by conclusions from the publication of Dewanto et al. (2002) [29], which is often cited in connection with increasing the content of polyphenols in cereals and deals with the effect of the cooking process on sweet corn. That study concluded that the increases in polyphenol content can be attributed to release of phenolic compounds from esterified or bound phenolic forms due to breakdown of the cellular constituents. Another contribution to TPC increase in the case of the cultivar Esmeralda could be the presence of hulls, because hulls are rich in these compounds and, in addition, they defend and prevent the loss of these compounds from grains during boiling. Duodu (2011) [13], in his review concerning the effect of processing on antioxidant phenolics, summarized a list of reasons which could lead to increases in phenolic content. Vukadinovic et al. (2022) [56] described that cooking significantly increased the concentration of protocatechuic, ferulic, and *p*-coumaric acids and decreased the concentrations of sinapic and caffeic acids in sweet corn. These authors also pointed out that increases or decreases in phenolic acids concentration depend upon their intracellular location and the plant tissue matrix, as well as upon the cooking method applied.

The results of this study showed increases in soluble phenolic content only in the case of two genotypes (i.e., AF Lucius and NMC), and this increase was not statistically significant. For the remaining four studied genotypes, decreases of 4–9% in soluble phenolic content were recorded (Table S2). The losses of phenolic compounds might be attributed to (i) their migration from the food matrix into the boiling water (leaching), (ii) their chemical degradation or transformation into other molecules, and (iii) their interaction with other food components that make them less extractable, as is described in the review from Duodu (2011) [13]. As far as the current study is concerned, the decreases in phenolic compounds due to leaching into water are less probable due to the reabsorption of the solution during boiling. On the other hand, the loss of soluble phenolic content could be lower when whole grains including brans are used, as was done in the current study, because this prevents leaching of the phenolic compounds during boiling. A significant decrease in polyphenol content in barley groats was reported by Krochmal-Marczak et al., 2019 [57]. They described that this may have resulted from the permeation of water-soluble polyphenols into the solution during boiling. Decreases in phenolic content were also registered in other studies focused on the effects of the boiling process on phenolic content in different species of cereals. The most similar cereal for which boiling is the typical thermal process is rice. The effect of boiling on phenolic compounds in rice has been described in several studies [23,25,58,59,60]. According to these studies, boiling different cultivars of rice resulted in reductions in phenolic content. Yu et al. (2021) [59] commented that pressure cooking preserved more phenols because normal boiling needs more time compared to pressure cooking. Those authors also concluded that the composition of phenolic acids after cooking remained unchanged compared to uncooked rice, but the contents of the individual phenolic acids were significantly different. Zaupa et al. (2015) [25] compared different domestic cooking procedures (“risotto” cooking and boiling) and found that risotto cooking, which allows for a complete absorption of water, retained more phenolic compounds. Other grains for which boiling is a typical thermal process include, among others, millet, fonio, sorghum, quinoa. In the cases of all the mentioned crops, boiling also resulted in reductions in their phenolic compound contents [27,38,61,62].

In the case of the insoluble phenols, decreases in phenolic content were registered in almost all genotypes. The greatest differences were found for NMC and AF Lucius, with decreases of around 1 mg GAE/g DM, which correspond to 9% and 11%, respectively (Table S2). Although the NMC genotype had one of the largest decreases in the insoluble phenols, it still maintained the highest content of insoluble phenols after boiling (10.53 mg GAE/g DM). All studied waxy-type genotypes had only minimal losses of insoluble phenols, ranging from 1% to 5%. According to the content of the insoluble phenols after boiling, the genotypes may be divided into three groups: NMC with the highest content, followed by the waxy-type genotypes, and finally by non-waxy-type barley genotypes.

If the sum of both the soluble and insoluble phenols is considered, a decrease by as much as 10% was registered for all genotypes after boiling. The highest value was obtained for NMC (12.66 mg GAE/g DM), and AF Cesar had the lowest (9.75 mg GAE/g DM). The remaining genotypes had values close together, and their mean was around 10.40 mg GAE/g DM (Table 1). Comparing the results in Table 1 makes clear that the non-waxy genotypes are less resistant to loss of phenolic compounds during boiling than are the waxy-type genotypes. The decrease in total phenolic content as observed may be due to some degradation of phenolic compounds, but supporting this assertion will require more analytical research. It has been reported, however, that phenolic acids exhibit better thermal stability than other phenolic compounds (e.g., anthocyanins or flavanols which were found in barley grains, as mentioned above) [63]. Zielinski et al. (2001) [37] noted that ferulic acid was dominant for barley after thermal processing.

Boiled grains not consumed fresh are usually kept in the refrigerator and later reheated, such as in a microwave oven. In this study, the procedure was the same, where as much as half of the boiled grains were kept in the refrigerator and then heated for 3 min in a microwave oven. The results are shown in the Table 1 and Table S2.

In the case of soluble phenols, the phenolic content decreased for all genotypes except AF Cesar, in which the content remained on the same level. This difference was not, however, statistically significant. Compared to grains that were only boiled (not reheated), the greatest decrease was found for NMC (13%), followed by genotype KM 2975 or KM 3189 (6%), AF Lucius (5%), and KM 2551 (3%) (Table S2).

For the insoluble polyphenols, decreases in content were observed across all genotypes. The greatest decrease, comparing boiled-only versus microwave-reheated grains, was again found for NMC and also KM 2975 (11%), for which the differences were statistically significant. They were followed by KM 3189 (9%), KM 2551 (6%), AF Lucius (4%), and AF Cesar (1%) (Table S2).

The sum of both the soluble and insoluble phenols in microwaved barley still showed a high value for the TPC. The highest value was obtained for NMC (11.20 mg GAE/g DM), and the lowest was obtained for KM 3189 (9.42 mg GAE/g DM). Except for NMC, all other genotypes had similar TPC values. Their mean, while excluding NMC, was 9.65 mg GAE/g DM, and there was no difference between the waxy and non-waxy types of barley.

The literature concerning the influence of microwave heating on total phenolic content of cereals is very limited. Sharma et al. (2011) [64] used eight barley varieties to study the effect of sand roasting and microwave cooking on TPC. The results showed that both cooking approaches lowered the TPC values. The publication written by Hithamani et al. (2014) [27] focuses on microwave heating of millet. Those authors stated that microwave heating did not significantly reduce the TPC of millet. Zhang et al. (2022) [65] described in their study that microwave heating significantly increased the TPC value for extracts from black quinoa. Zhang et al. (2010) [66] studied the effects of different thermal processing methods, including microwave heating, on whole-meal flour from buckwheat. Their results showed a decrease in the phenolic content after microwave heating, which was explained as a consequence of phenolic destruction during heating. Reductions in phenolic compounds content, regardless of genotype, were also recorded in the current study, although the procedure was completely different.

The total losses caused by both thermal processes (boiling and microwave heating) ranged from 10% to 17% (Table S2). The largest decrease was recorded for NMC, so we can assume that this genotype had the weakest resistance to thermal processing. On the other hand, waxy genotypes KM 3189 and KM 2551, together with genotype AF Cesar, had the smallest losses.

### 3.2. Antioxidant Activity

Antioxidant activity (AA) is defined as the ability of a chemical compound to stabilize or neutralize the oxidative action of free radicals. In the case of barley, phenolic compounds are considered to comprise the major group of compounds contributing to this property [67]. The AA capacities of individual phenolic compounds depend on their structure and configuration, such as the number and position of the hydroxyl groups, as well as the character of substituents and their positions in relation to the hydroxyl groups [68]. The term “antioxidant activity”, in this study, corresponds to the cumulative action of all the antioxidants in the extract expressed as an equivalent to Trolox activity. In the current study, two methods were used to measure the AA of the samples: (i) DPPH assay and (ii) TEAC assay. Both methods are commonly used to evaluate the AA of plant extracts and are based on chromogen radicals that undergo colour change upon reduction by antioxidant [69].

For both DPPH and TEAC assays, the results showed substantial AAs whether for soluble or insoluble phenolic compounds. The results are listed in Table 2, Tables S3 and S4.

For soluble phenolic compounds (Table 2), the TEAC method gave higher values, but both methods showed similar results in natural grains regarding the order of genotypes. AF Lucius had the highest AA, at 4.02 mg TE/g DM for the DPPH and 7.82 mg TE/g DM for the TEAC method. Next was AF Cesar (3.55 mg TE/g DM for DPPH and 6.76 mg TE/g DM for TEAC). The lowest value was recorded for NMC (3.23 mg TE/g DM for DPPH and 5.45 mg TE/g DM for TEAC). The results for waxy-type barley genotypes were grouped closely together. In the case of the DPPH method, the mean was 3.42 mg TE/g DM, and for the TEAC method, the results were varying around 6.0 mg TE/g DM.

Lin at al., 2018 [70] had studied phenolic profiles and AAs of soluble phenolic compounds in three Tibetan hull-less barley varieties with different colours. They found DPPH scavenging activity between 2.28 and 2.78 mg TE/g DM and TEAC values ranging from 1.75 to 1.97 mg TE/g DM. Such values are lower than those of the current study, but this may be explained by the different genotypes studied and a different approach to extraction. In the current study, the TEAC method gave values higher than did the DPPH assay. This trend was also described in the study of Floegel et al., 2011 [71]. High AA (as measured by both the DPPH and TEAC assays) in natural barley grains has been reported also by Zhao et al., 2008 [51], who studied the AA of malting barley varieties. They found significant differences in the AA between individual varieties in the results of the two methods. Differences in the AA between individual varieties have been explained by dissimilar phenolic compositions. Different phenolic compositions of barley genotypes were clearly shown in the study of Martinez et al., 2018 [72], who examined the phytochemical compositions of 27 barley genotypes that included hulled and hull-less varieties. Among other things, they analysed 64 phenolic compounds, including phenolic acids, flavones, and anthocyanins. Comparatively, 5 of 27 genotypes from their study (AF Cesar, AF Lucius, NMC, KM 2975, and KM 2551) can also be found in the current work.

After boiling, the DPPH method determined that the AA values had increased for almost all genotypes except for AF Cesar, for which there was an 8% decrease (Table S3). However, almost no changes were statistically significant. The exception was NMC, for which the largest increase (15%) was recorded. This black genotype was followed by KM 2975 (10%) and KM 2551 (8%), and the smallest increases were for KM 3189 and AF Lucius at 3% and 2%, respectively. Although the variety AF Lucius had the smallest increase in AA, it maintained the highest antioxidant value after boiling. AF Cesar had the lowest AA. For the TEAC method, a statistically significant decrease in AA was recorded for AF Cesar (18%) and AF Lucius (11%) (Table S4). A statistically non-significant increase was found for NMC (8%), and the waxy genotypes had AA values comparable to that of the natural grain.

Microwave heating also resulted in some changes in AA. Using the DPPH method, increases were recorded for four genotypes. The greatest increase was for genotype KM 3189 (10%), followed by AF Cesar and KM 2551 (6%). AF Lucius showed the smallest increase at 4%. For NMC and KM 2975, decreases in AA were registered at 4% and 3%, respectively. However, none of these changes were significant. Using the TEAC method, subsequent microwave heating caused the increase of AA for all genotypes except KM 2975, for which the activity decreased by about 3%, but again, these changes were not statistically significant. AF Cesar showed the largest increase, at 9%, followed by NMC, with 4%. The results for waxy genotypes did not change significantly (2%), remaining around 6.0 mg TE/g DM.

Upon summarizing the results for the soluble polyphenols, the variety AF Lucius kept the highest activity for soluble phenols through both thermal processes independently of which measurement method was used. In the case of the DPPH method, the boiling and subsequent microwave heating led to growths in AA for almost all genotypes. The greatest increases were recorded for KM 2551, KM 3189, and NMC (>10%). For the TEAC method, the results were not so clear as for the DPPH method, as the three genotypes demonstrated increases in activity, and for three, declines were registered. NMC had the largest gain in AA (12%), and the greatest decrease in activity (11%) was found for AF Cesar. The AAs of the waxy-type genotypes did not change significantly.

In reviewing the literature concerning the cooking of different cereals, one may get the impression that decreases in AA as measured by various methods are more probable. For barley, there is a study from Gallegos-Infante et al. (2010) [35] describing decrease in AA as determined by DPPH. The DPPH method was also used in a study from Hes et al. (2014) [73], who examined the effect of boiling on barley and buckwheat groats. They also registered decreases in AA. Additionally, AA reductions were described by several authors studying thermal treatments of other cereals, including rice [21,22,23,25,58,59,74,75], buckwheat [57,64,73,76,77], millet [28,38,57], and sorghum [38]. Reduced AA can be attributed to several factors, with the main among these being (i) water volume used for cooking, because phenolic compounds can be leached into this water; (ii) temperature, which may influence the stability of some compounds (e.g., anthocyanins); and (iii) cooking time [78]. On the other hand, an increase in AA was recorded in a publication from Dewanto et al. (2002) [29], who described an increase in AA (measured by total oxyradical scavenging activity) for sweet corn cooked in different conditions (varying heating time and temperature). Fracassetti et al. (2020) [78] studied several cooking methods (boiling, microwaving, pressure cooking, water bath, and rice cooker) on pigmented rice cultivars. In the case of cooking in laboratory conditions (cooking in water bath for 40 min; water: rice ratio was 2:1), they registered by the DPPH method increases in AA for three tested genotypes. On the contrary, the AA values measured by TEAC assay showed decreases in activity for two of three genotypes and for the final 1 value was unchanged. The difference in results obtained by the two methods was ascribed to decreases in the amounts of certain phenolics, namely, procyanidins, that are preferentially bound with ABTS cation radical [79]. Other cooking methods caused decreases in activity, as measured by both approaches.

The literature, which is not very abundant on the subject, describes both increases and decreases in AA after microwave heating. E.g. Sharma et al. (2011) [64] roasted eight different barley varieties in a microwave oven (120 s at 900 W) and analysed AA by DPPH assay. Their results showed increases in AA in all barley varieties, ranging from 16.8% to 80.2%. Omwaba et al. (2009) [80] tried to optimise the condition of roasting barley grains in a microwave oven using the response surface methodology to obtain grains with high AA. They concluded that the DPPH radical scavenging activity of the sample increased significantly with an increase in the power of the microwave oven. Zhang et al. (2022) [65] described in their study how different cooking methods (boiling, roasting, and microwaving) influence the AA of black quinoa measured by DPPH and TEAC methods. They stated that microwave heating significantly improved the AA determined by both assays. On the other hand, a decrease in the AA of maize was described by Jiménez-Monreal et al. (2009) [81], who studied the influence of home cooking methods (including, among others, boiling, microwaving, and baking) on AA. The effect of microwave heating on AA of tartary buckwheat flour was studied by Zhang et al. (2010) [66]. They found out that the scavenging activity of hydroxyl radical decreased from 93.13% to 30.63% after the buckwheat was microwave-heated.

In current study, the AA values of samples after two cooking steps measured by DPPH assay increased for almost all genotypes (the exception being AF Cesar), but the differences were significant only for three of them (NMC, KM 3189 and KM 2551). For the TEAC method, all waxy type genotypes had results close together, and there was no significant difference between raw and thermally treated samples with regard to AA. Statistically significant were reductions in AA for the varieties AF Cesar and AF Lucius and an increase in activity for NMC. The differences between the results obtained by the two approaches may be ascribed to different phenolic composition of the individual genotypes, as described above, because the AA is affected not only by quantity but also by the kind of free radical scavengers present in extract [82]. Other reason for such difference may be differences in reaction kinetics between those phenols present in extract and which radical (DPPH or TEAC) is used [83].

In the case of insoluble phenolic compounds (Table 2), the evaluation by TEAC method resulted in nearly 10 times higher AA values than those from the DPPH method. For the DPPH method, the genotypes could be ordered into three groups according to their AA in natural grain, which means NMC (7.76 mg TE/g DM) > AF Lucius ≈ AF Cesar (6.72 mg TE/g DM and 6.50 mg TE/g DM) > KM 2975 ≈ KM 3189 ≈ KM 2551 (5.79 mg TE/g DM, 5.55 mg TE/g DM, and 5.37 mg TE/g DM). In the case of the TEAC method, the mean for all genotypes together in raw grains was 52.14 mg TE/g DM, and the differences were not statistically significant.

For the DPPH method, boiling caused an increase in AA for waxy-type lines of barley and a decrease for the rest of the genotypes. The black NMC had the highest AA (6.99 mg TE/g DM), despite the decline of around 10% after boiling. The AA for NMC after boiling was statistically comparable with the activity of the waxy-type barley genotypes; however, which followed with values around 6.20 mg TE/g DM. In their cases, there were increases in AA by 8%, 11%, and 16% for KM 2975, KM 3189, and KM 2551, respectively. For AF Lucius and AF Cesar, the AA values decreased after boiling by more than 30%, and thus they had the lowest AA values of insoluble polyphenols as measured by DPPH. In the case of the TEAC method, the results were different. There was a registered slight increase of around 3% in the AA for almost all varieties except NMC, for which the increase was negligible. NMC still remained the genotype with the strongest activity, followed by the waxy genotypes. The lowest activity was in varieties AF Cesar and AF Lucius.

Subsequent microwave heating resulted in a decline in the AA of insoluble phenols for all barley genotypes measured by the TEAC method. The highest value was recorded for NMC (52.29 mg TE/g DM), but the decrease in activity compared to boiled grains was lowest (7%). For the remaining genotypes, a decline of more than 11% was registered. The lowest activity was found for KM 2975 (46.38 mg TE/g DM). For the DPPH method, the AA of insoluble phenols was also highest for NMC (6.71 mg TE/g DM). The decrease in activity compared to boiled grains was 4%. The strongest decline, measured by the DPPH method, was found for KM 2975, followed by KM 3189, at 14% and 10%, respectively. In the case of varieties AF Lucius and AF Cesar, the AA increased by 7% and 6%, respectively, but both had the lowest activity compared to the rest of the genotypes.

When considering the combined effects on insoluble phenolic compounds of the two steps of the thermal process together, the AA decreased for almost all genotypes regardless of the measurement method used. In the case of DPPH, the greatest decrease was registered for varieties AF Lucius and AF Cesar (around 41%). For these varieties, the reduction was not so strong in the case of the TEAC method. NMC had kept the highest AA during both thermal processes whether measured by DPPH or TEAC. AF Cesar and AF Lucius had the lowest activity as measured by DPPH and genotype KM 2975 as measured by TEAC.

## 4. Conclusions

Six different hull-less spring barley genotypes intended for prospective human nutrition were tested for polyphenol content, antioxidant activity, and the influence of kitchen preparation. All tested genotypes were proven to be good sources of phenolic compounds, with no significant differences between waxy (10.73 ± 0.22 mg GAE/g DM) and non-waxy (11.83 ± 1.24 mg GAE/g DM) barley types. Thermal processes commonly used in the grain’s preparation for human consumption, such as boiling and subsequent microwave heating, reduced the phenolic content and antioxidant activity to some extent. After both process steps, the decline in the total content of phenolic compounds was significant for all genotypes and ranged between 10 and 17%. Antioxidant activity of soluble and insoluble phenolic compounds differed as analysed by TEAC and DPPH methods, with TEAC giving higher values, especially in the case of insoluble phenolic compounds. In the case of raw grain, the mean of all genotypes as analysed by TEAC was 52.14 ± 1.62 TE/g DM, as analysed by DPPH 6.28 ± 0.82 TE/g DM.

The measured response to thermal treatment also differed according to the method used. After both process steps, a significant increase of soluble polyphenols in three of six genotypes and a significant decrease of insoluble polyphenols in two of six genotypes was observed using the DPPH method, but using the TEAC method, a decrease was observed for both soluble (in three of six genotypes) and insoluble (in two of six genotypes) polyphenols. The results confirmed that despite some reduction after the kitchen preparation, food based on barley could be an important source of polyphenols in human nutrition. If we take into account the other substances present in hull-less barley grain, such as β-glucans, vitamins, and minerals, it could be suggested that barley deserves a more important role in the human diet.

## Figures and Tables

**Table 1:** Total phenolic content (TPC) in grains of six hull-less barley genotypes before and after two steps of heat treatment (boiling and subsequent microwave heating). Values are expressed as mg of gallic acid equivalent (GAE) in gram of dry matter (mg GAE/g DM). Lowercase letters indicate statistically significant differences (*p* < 0.05) between individual genotypes (columns). Additionally, uppercase letters indicate statistical differences for individual heat treatment steps within the studied genotypes (rows).

Phenolic Group	Genotype	TPC (mg GAE/g DM)		
		Raw	Boiled	Boiled and Microwaved
**Soluble**				
	AF Cesar	2.23 ± 0.04 ^bA^	2.03 ± 0.08 ^bA^	2.03 ± 0.07 ^bcA^
	AF Lucius	2.52 ± 0.05 ^aA^	2.55 ± 0.04 ^aA^	2.42 ± 0.07 ^aA^
	NMC	2.04 ± 0.02 ^cAB^	2.13 ± 0.12 ^bA^	1.85 ± 0.07 ^cB^
	KM 2975	2.19 ± 0.02 ^bA^	2.10 ± 0.09 ^bA^	1.97 ± 0.06 ^bcA^
	KM 3189	2.22 ± 0.02 ^bA^	2.13 ± 0.07 ^bA^	2.00 ± 0.14 ^bcA^
	KM 2551	2.25 ± 0.02 ^bA^	2.14 ± 0.09 ^bA^	2.08 ± 0.08 ^bA^
	**Mean**	**2.24 ± 0.14 ^A^**	**2.18 ± 0.17 ^B^**	**2.06 ± 0.17 ^C^**
**Insoluble**				
	AF Cesar	8.51 ± 0.23 ^bA^	7.72 ± 0.08 ^cAB^	7.63 ± 0.24 ^bB^
	AF Lucius	8.66 ± 0.42 ^bA^	7.74 ± 0.25 ^cB^	7.40 ± 0.10 ^bB^
	NMC	11.51 ± 0.24 ^aA^	10.53 ± 0.41 ^aB^	9.35 ± 0.69 ^aC^
	KM 2975	8.69 ± 0.24 ^bA^	8.53 ± 0.22 ^bA^	7.58 ± 0.40 ^bB^
	KM 3189	8.20 ± 0.24 ^bA^	8.15 ± 0.26 ^bcA^	7.42 ± 0.25 ^bA^
	KM 2551	8.64 ± 0.18 ^bA^	8.20 ± 0.40 ^bcAB^	7.72 ± 0.28 ^bB^
	**Mean**	**9.04 ± 1.12 ^A^**	**8.48 ± 0.96 ^B^**	**7.85 ± 0.68 ^C^**
**Total**				
	AF Cesar	10.74 ± 0.25 ^bcA^	9.75 ± 0.01 ^bB^	9.66 ± 0.26 ^bB^
	AF Lucius	11.18 ± 0.45 ^bA^	10.29 ± 0.27 ^bAB^	9.82 ± 0.09 ^bB^
	NMC	13.55 ± 0.23 ^aA^	12.66 ± 0.52 ^aA^	11.20 ± 0.72 ^aB^
	KM 2975	10.88 ± 0.25 ^bcA^	10.63 ± 0.29 ^bA^	9.55 ± 0.42 ^bB^
	KM 3189	10.42 ± 0.26 ^cA^	10.28 ± 0.26 ^bAB^	9.42 ± 0.34 ^bB^
	KM 2551	10.89 ± 0.20 ^bcA^	10.34 ± 0.43 ^bAB^	9.80 ± 0.31 ^bB^
	**Mean**	**11.28 ± 1.04 ^A^**	**10.66 ± 0.93 ^B^**	**9.91 ± 0.59 ^C^**

**Table 2:** Antioxidant activity results of barley grains before and after thermal processing as measured by DPPH and TEAC methods. Values are expressed as milligrams of Trolox equivalent (TE) per gram of dry matter (mg TE/g DM). Lowercase letters indicate statistically significant differences (*p* < 0.05) between individual genotypes (columns). Additionally, uppercase letters indicate statistical differences for individual heat treatment steps within the studied genotypes (rows).

Genotype	DPPH			TEAC		
	Raw	Boiled	Boiled and Microwaved	Raw	Boiled	Boiled and Microwaved
**Soluble**						
AF Cesar	3.55 ± 0.03 ^bA^	3.26 ± 0.12 ^dA^	3.45 ± 0.14 ^cA^	6.76 ± 0.16 ^bA^	5.53 ± 0.27 ^bB^	6.05 ± 0.17 ^bB^
AF Lucius	4.02 ± 0.04 ^aA^	4.12 ± 0.13 ^aA^	4.28 ± 0.07 ^aA^	7.82 ± 0.10 ^aA^	6.92 ± 0.26 ^aB^	7.09 ± 0.14 ^aB^
NMC	3.23 ± 0.07 ^cB^	3.73 ± 0.10 ^bcA^	3.59 ± 0.11 ^bcA^	5.45 ± 0.11 ^dB^	5.89 ± 0.18 ^bAB^	6.12 ± 0.16 ^bA^
KM 2975	3.48 ± 0.03 ^bA^	3.82 ± 0.11 ^abA^	3.69 ± 0.06 ^bcA^	5.98 ± 0.19 ^cA^	6.04 ± 0.27 ^bA^	5.86 ± 0.25 ^bA^
KM 3189	3.29 ± 0.04 ^cB^	3.40 ± 0.18 ^cdAB^	3.72 ± 0.21 ^bcA^	6.01 ± 0.03 ^cA^	5.93 ± 0.04 ^bA^	6.05 ± 0.15 ^bA^
KM 2551	3.50 ± 0.04 ^bB^	3.78 ± 0.14 ^abcAB^	4.00 ± 0.25 ^abA^	6.04 ± 0.09 ^cA^	5.98 ± 0.05 ^bA^	6.09 ± 0.30 ^bA^
**Mean**	**3.51 ± 0.25 ^A^**	**3.68 ± 0.28 ^B^**	**3.79 ± 0.27 ^C^**	**6.34 ± 0.76 ^A^**	**6.05 ± 0.42 ^C^**	**6.21 ± 0.40 ^B^**
**Insoluble**						
AF Cesar	6.50 ± 0.19 ^bcA^	4.43 ± 0.43 ^bB^	4.69 ± 0.09 ^cB^	51.86 ± 2.97 ^aAB^	52.88 ± 1.99 ^aA^	46.87 ± 0.56 ^bB^
AF Lucius	6.72 ± 0.29 ^bA^	4.53 ± 0.28 ^bB^	4.84 ± 0.13 ^cB^	51.02 ± 2.83 ^aAB^	52.64 ± 0.81 ^aA^	46.74 ± 1.07 ^bB^
NMC	7.76 ± 0.65 ^aA^	6.99 ± 0.27 ^aA^	6.71 ± 0.64 ^aA^	55.72 ± 1.09 ^aA^	55.98 ± 2.67 ^aA^	52.29 ± 1.28 ^aA^
KM 2975	5.79 ± 0.24 ^cdA^	6.23 ± 0.32 ^aA^	5.37 ± 0.30 ^bcA^	51.29 ± 2.33 ^aA^	52.97 ± 2.13 ^aA^	46.38 ± 0.66 ^bB^
KM 3189	5.55 ± 0.37 ^dA^	6.18 ± 0.40 ^aA^	5.53 ± 0.43 ^bcA^	51.52 ± 1.29 ^aA^	53.05 ± 1.76 ^aA^	46.88 ± 1.10 ^bB^
KM 2551	5.37 ± 0.08 ^dA^	6.22 ± 0.48 ^aA^	5.97 ± 0.45 ^abA^	51.45 ± 0.99 ^aAB^	53.24 ± 0.99 ^aA^	47.53 ± 1.67 ^bB^
**Mean**	**6.28 ± 0.82 ^A^**	**5.76 ± 0.95 ^B^**	**5.52 ± 0.68 ^B^**	**52.14 ± 1.62 ^B^**	**53.46 ± 1.14 ^A^**	**47.78 ± 2.04 ^C^**

## Data Availability

The original contributions presented in the study are included in the article/Supplementary Materials, further inquiries can be directed to the corresponding author.

## Supplementary Materials

The following supporting information can be downloaded at https://www.mdpi.com/article/10.3390/foods14152578/s1. Table S1: Dry weight of grain of six hull-less barley varieties before and after boiling and subsequent microwave heating. Table S2: Differences in TPC values before and after individual steps of preparation. The results are expressed in %. The negative value indicates a decrease in TPC. Asterisk (*) indicates statistically significant change. Table S3: Differences in values of the antioxidant activity, measured by the DPPH method, before and after individual steps of preparation. The results are expressed in %. The negative value indicates a decrease in antioxidant activity. Asterisk (*) indicates statistically significant change. Table S4: Differences in values of the antioxidant activity, measured by the TEAC method, before and after individual steps of preparation. The results are expressed in %. The negative value indicates a decrease in antioxidant activity. Asterisk (*) indicates statistical different change.

## Abbreviations

AA	Antioxidant activity
ABTS	2,2′-Azinobis (3-ethylbenzothiazoline-6-sulfonic acid) diammonium salt
DPPH	2,2-diphenyl-1-pycrylhydrazyl radical
NMC	Nudimelanocrithon
TEAC	Trolox equivalent antioxidant activity
TPC	total polyphenol content
