# Supplementary material for: Effect of Domestic Cooking of Hull-Less Barley Genotypes on Total Polyphenol Content and Antioxidant Activity
Source: Foods. 2025 Jul 23;14(15):2578. doi: 10.3390/foods14152578 (PMC12345685; doi:10.3390/foods14152578)
Supplement: Supplementary file 1 [file foods-14-02578-s001.zip › foods-3693445-supplementary.pdf]

Table S1: Dry weight (DW) of six hull-less barley samples before and after boiling and subsequent microwave heating.

Cultivar	Dry weigh (%)		
	Raw	Boiled	Boiled and Microwaved
AF Cesar	90.7	53.0	49.8
AF Lucius	90.3	52.9	54.3
NMC	89.5	61.2	58.5
KM 2975	89.6	54.5	53.8
KM 3189	90.6	53.4	51.4
KM 2551	90.9	54.1	51.3

Table S2: TPC differences between obtained values before and after individual steps of preparation. The results are expressed in %. The negative value indicates a decrease in TPC. Star (*) indicates statistically significant change.

	Cultivar	TPC differences (%)		
		Raw – Boiled	Boiled – Boiled and Microwaved	Raw – Boiled and Microwaved
**Soluble**				
	AF Cesar	-9	0	-9
	AF Lucius	1	-5	-4
	NMC	4	-13*	-9
	KM 2975	-4	-6	-10
	KM 3189	-4	-6	-10
	KM 2551	-5	-3	-7
	**Mean**	**-3***	**-6***	**-8***
**Insoluble**				
	AF Cesar	-9	-1	-10*
	AF Lucius	-11*	-4	-15*
	NMC	-9*	-11*	-19*
	KM 2975	-2	-11*	-13*
	KM 3189	-1	-9	-10
	KM 2551	-5	-6	-11*
	**Mean**	**-6***	**-7***	**-13***
**Total**				
	AF Cesar	-9*	0	-10*
	AF Lucius	-8	-5	-12*
	NMC	-7	-12*	-17*
	KM 2975	-2	-10*	-12*
	KM 3189	-1	-8	-10*
	KM 2551	-5	-5	-10*
	**Mean**	**-5***	**-7***	**-12***

Table S3: Differences between obtained values of the antioxidant activity, measured by the DPPH method, before and after individual steps of preparation. The results are expressed in %. The negative value indicates a decrease in antioxidant activity. Star (*) indicates statistically significant change.

	**Cultivar**	**Differences (%)**		
		Raw – Boiled	Boiled – Boiled and Microwaved	Raw – Boiled and Microwaved
**Soluble**				
	AF Cesar	-8	6	-3
	AF Lucius	2	4	6
	NMC	15*	-4	11*
	KM 2975	10	-3	6
	KM 3189	3	10	13*
	KM 2551	8	6	14*
	**Mean**	**5***	**3**	**8**
**Insoluble**				
	AF Cesar	-32*	6	-41*
	AF Lucius	-33*	7	-41*
	NMC	-10	-4	-15
	KM 2975	8	-14	-7
	KM 3189	11	-10	0
	KM 2551	16	-4	10
	**Mean**	**-8**	**-4**	**-13**

Table S4: Differences between obtained values of the antioxidant activity, measured by the TEAC method, before and after individual steps of preparation. The results are expressed in %. The negative value indicates a decrease in antioxidant activity. Star (*) indicates statistically significant change.

Cultivar	Differences (%)		
	Raw – Boiled	Boiled – Boiled and Microwaved	Raw – Boiled and Microwaved
**Soluble**			
AF Cesar	-18*	9	-11*
AF Lucius	-11*	2	-9*
NMC	8	4	12*
KM 2975	1	-3	-2
KM 3189	-1	2	1
KM 2551	-1	2	1
**Mean**	**-5**	**3**	**-2**
**Insoluble**			
AF Cesar	2	-11*	-10
AF Lucius	3	-11*	-8
NMC	0	-7	-6
KM 2975	3	-12*	-10*
KM 3189	3	-12*	-9*
KM 2551	3	-11*	-8
**Mean**	**3**	**-11**	**-8**
